# A combination of serum iron, ferritin and transferrin predicts outcome in patients with intracerebral hemorrhage

**DOI:** 10.1038/srep21970

**Published:** 2016-02-22

**Authors:** Guang Yang, Rong Hu, Chao Zhang, Christopher Qian, Qian-Qian Luo, Wing-Ho Yung, Ya Ke, Hua Feng, Zhong-Ming Qian

**Affiliations:** 1 Department of Neurosurgery, South-west Hospital, The Third Military Medical University, 30 Gaotanyan Street, Shapingba District, Chongqing 400038, China; 2 School of Biomedical Sciences, Faculty of Medicine, the Chinese University of Hong Kong, Shatin, NT, Hong Kong SAR, China; 3 Laboratory of Neuropharmacology, Fudan University School of Pharmacy, Shanghai 201203, PRC

## Abstract

Association of a high-serum ferritin with poor outcome showed that iron might play a detrimental role in the brain after intracerebral hemorrhage (ICH). Here, we investigated changes in serum iron, ferritin, transferrin (Tf) and ceruloplasmin (CP) in patients with ICH (n = 100) at day 1 (admission), 3, 7, 14 and 21 and those in control subjects (n = 75). The hematoma and edema volumes were also determined in ICH-patients on admission and at day 3. The Modified Rankin Scale (mRS) of 59 patients was ≥3 (poor outcome) and 41 < 3 (good outcome) at day 90. Serum ferritin was significantly higher and serum iron and Tf markedly lower in patients with poor-outcome than the corresponding values in patients with good-outcome at day 1 to 7 and those in the controls. There was a significant positive correlation between serum ferritin and relative edema volume or ratio at day 1 and 3 and hematoma volume at day 1 (n = 28), and a negative correlation between serum iron or Tf and hematoma volume at day 1 (n = 100). We concluded that not only increased serum ferritin but also reduced serum iron and Tf are associated with outcome as well as hematoma volume.

Intracerebral hemorrhage (ICH) accounts for about 10% to 15% of strokes in Western countries and up to 20% to 30% in Asian countries^1^, affecting more than one million people worldwide annually^2^,^3^. ICH occurs when a blood vessel in part of the brain becomes weak and bursts open, causing blood to leak into the brain^4^,^5^ and is associated with higher mortality and worse clinical outcome than ischemic stroke, and no effective therapy is available currently^1^,^4^,^6^.

Evidence indicates that iron plays a key role in mediating neuronal injury and edema formation after ICH^7^,^8^. Iron, a hemoglobin degradation product after erythrocyte lysis and also released from ferritin stores, is neurotoxic by catalyzing hydroxyl radical formation and promoting oxidative stress^7^,^9^. *In vivo* studies in animal models of ICH also demonstrate that iron accumulates in the brain after ICH^7^ and iron-modifying agents can reduce hemoglobin-induced neurotoxicity and brain edema^10^. Clinical studies observe that serum ferritin, a reliable indicator of body iron load^11^,^12^ is associated with relative perihematoma edema volume^8^,^13^,^14^ and is an important predictor of clinical outcome in patients with ICH^15^,^16^.

To better understand the link between iron and neuronal injury after ICH and the effect of iron on functional outcome of ICH, it is necessary to have a more detailed picture of the changes in iron metabolism induced by ICH. Although a number of experimental studies to investigate this topic have been conducted, few data exist about clinical investigations of this issue. At present, there was not much information on the effects of ICH on serum iron and transferrin (Tf), a key iron-transport-protein and ceruloplasmin (CP), an acute phase reactant in ICH patients. In the present study, we therefore investigated the changes in serum iron, ferritin, Tf and CP in 100 consecutive patients with acute spontaneous ICH during the period from admission to day 21 and also measured hematoma and edema volumes on admission and at day 3. In addition, we used Spearman correlation coefficient to determine the association of these indicators of iron metabolism we measured with relative edema volume, relative edema ratio and hematoma volume as well as serum iron with ferritin, CP or Tf, and ferritin with Tf contents.

## Results

### Baseline characteristics

A total of 104 patients were admitted to the stroke unit of our department during the study period. Four subjects were excluded from the present analysis; 1 aged over 80, and 1 had a secondary cause for ICH (vascular malformations) and 2 did not have a follow-up computed tomographic (CT) because of withdrawal of care. Data from the remaining 100 subjects, who met all of our inclusion and none of our exclusion criteria, were included. The Modified Rankin Scale (mRS) of 59 patients was more than or equals to 3 (PO, poor outcome) and 41 less than 3 (GO, good outcome) at 90 days following treatment initiation. Table 1 listed the Baseline Characteristics of clinical, biochemical and radiological investigations by outcome groups. The mean age was 56 years (range 40 to 80). Seventy-two percent were men, and twenty-eight percent women. Higher stroke severity, lower platelet concentration in the blood, deep location of hematoma, subtentorial hematoma and intraventricular extension of hemorrhage at baseline were associated with poor clinical outcome.

### Changes in serum iron, ferritin, transferrin and ceruloplasmin in ICH patients

#### General tendency

**Figure 1:**
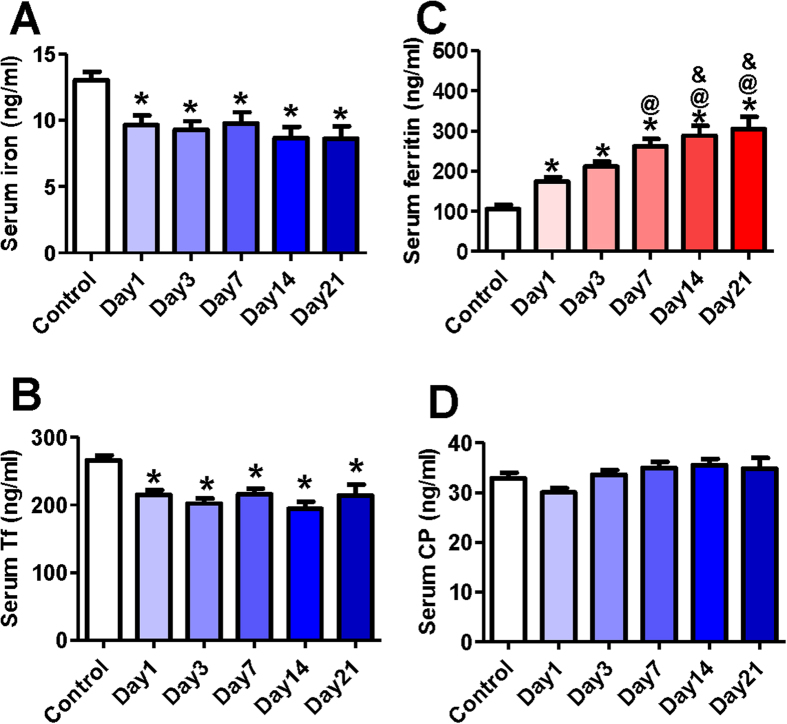
Serum iron, ferritin, transferrin and ceruloplasmin contents in patients with acute intracerebral hemorrhage (ICH). Serum samples were taken immediately from the control (n = 75) and ICH patients on admission (Day 1, n = 100), at day 3 (n = 91), 7 (n = 78), 14 (n = 52) and 21 (n = 30) after admission. And then the contents of iron (**A**), transferrin (**B**), ferritin (**C**) and ceruloplasmin (**D**) were determined as described in methods section. Data were presented as Mean ± SD. *P < 0.05 vs. Control; ^**@**^P < 0.05 vs. Day 1; ^**&**^P < 0.05 vs. Day 3.

Figure 1 showed the contents of serum iron, ferritin, Tf and CP in all subjects with ICH at all time-points. Serum iron (Fig. 1A) and Tf (Fig. 1B) levels at day 1 (baseline) of ICH patients were found to be significantly lower than those in the control. This implied that ICH could lead to a significant reduction in serum iron and Tf. The levels of iron and Tf after ICH were in a relatively stable state, no significant differences in these two markers being found between day 1 and day 3 or 7, 14 and 21. In the case of ferritin, the levels at day 1 were significantly higher than those in the control, evidencing a significant increase in serum ferritin induced by ICH (Fig. 1C). It was also found that with the time, the contents of ferritin in serum progressively increased, there being significant differences among these time-points. No significant differences were found in serum CP between the control and day 1 or any other time-points (Fig. 1D).

#### Iron, transferrin, ferritin and ceruloplasmin in serum in ICH patients with poor- and good-outcome

**Figure 2:**
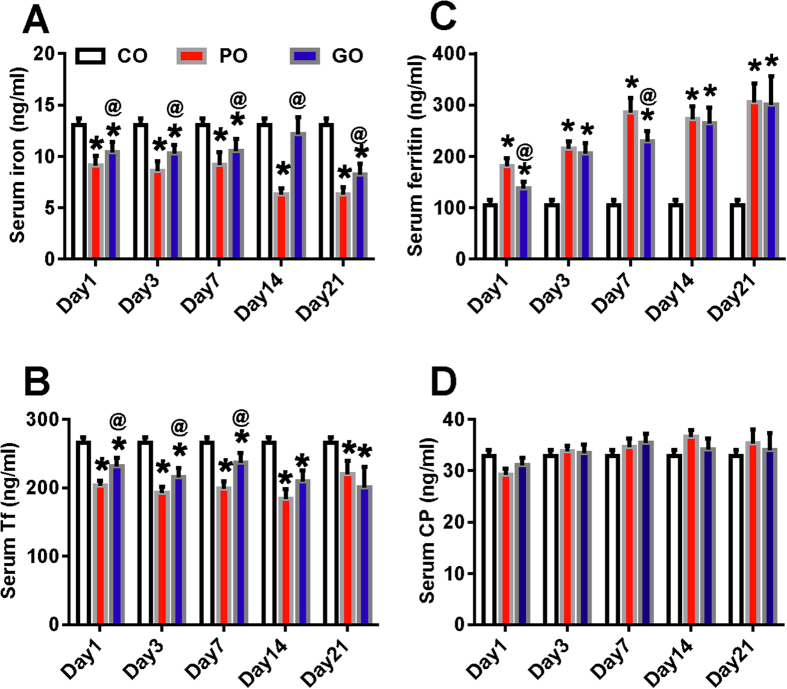
The lower iron and transferrin contents and the higher ferritin levels in serum in ICH patients with poor-outcome. Serum samples were taken immediately from the control (n = 75) and ICH patients on admission (day 1), at day 3, 7, 14 and 21 after admission. And then the contents of iron (**A**), transferrin (**B**), ferritin (**C**) and ceruloplasmin (**D**) were determined as described in methods section. n = 59 (day 1), 53 (day 3), 44 (day 7), 31(day 14) and 21 (day 21) in PO group, and n = 41 (day 1), 38 (day 3), 34 (day 7), 21 (day 14) and 9 (day 21) in good-outcome (GO) group.

**Figure 3:**
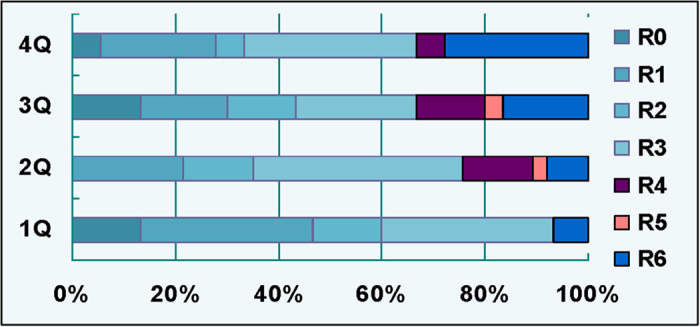
Distribution of modified Rankin scale scores at 90 days by serum ferritin levels categorized in quartiles. Ferritin levels for the first quartile (**1Q**), 0–80 ng/mL; second quartile (**2Q**), 81–160 ng/mL; third quartile (**3Q**), 161–275 ng/mL; fourth quartile (**4Q**), >276 + ng/ml. Data were presented as Mean ± SD. *P < 0.05 vs. Control; ^@^P < 0.05 vs. PO.

We then compared the levels of these four indicators of iron metabolism in patients with the poor- and good outcomes (Fig. 2). It was found that serum iron levels in patients with poor-outcome were significantly lower than those in the control as well as in the patients with good-outcome at all time-points. Also, there were significantly differences in serum iron levels between the patients with good-outcome and the control at day 1, 3, 7 and 14. (Fig. 2A). The contents of serum Tf in patients with poor- and good-outcomes both were significantly lower than those in the control, however, significance in the difference in serum Tf between the patients with good- and poor-outcomes was found only at day 1, 3 and 7 (Fig. 2B). In contrast to levels of iron and Tf in serum, ferritin contents in the patients with poor- and good-outcome both were significantly higher than those in the control. The patients with poor-outcome have a significant higher content of ferritin as compared with the patients with good-outcome at day 1 and 7, but not at day 3, 14 and 21 (Fig. 2C). No differences in serum CP were found among the control, poor- and good-outcome groups (Fig. 2D). Figure 3 showed the distribution of modified Rankin scale scores at 90 days by serum ferritin levels categorized in quartiles. It was found that there was a relationship between the distribution of the mRS and baseline levels of ferritin categorized in quartiles.

#### Effects of intraventricular hemorrhage, hematoma volumes and surgery treatment on the contents of iron, ferritin, transferrin and ceruloplasmin in the serum

**Figure 4:**
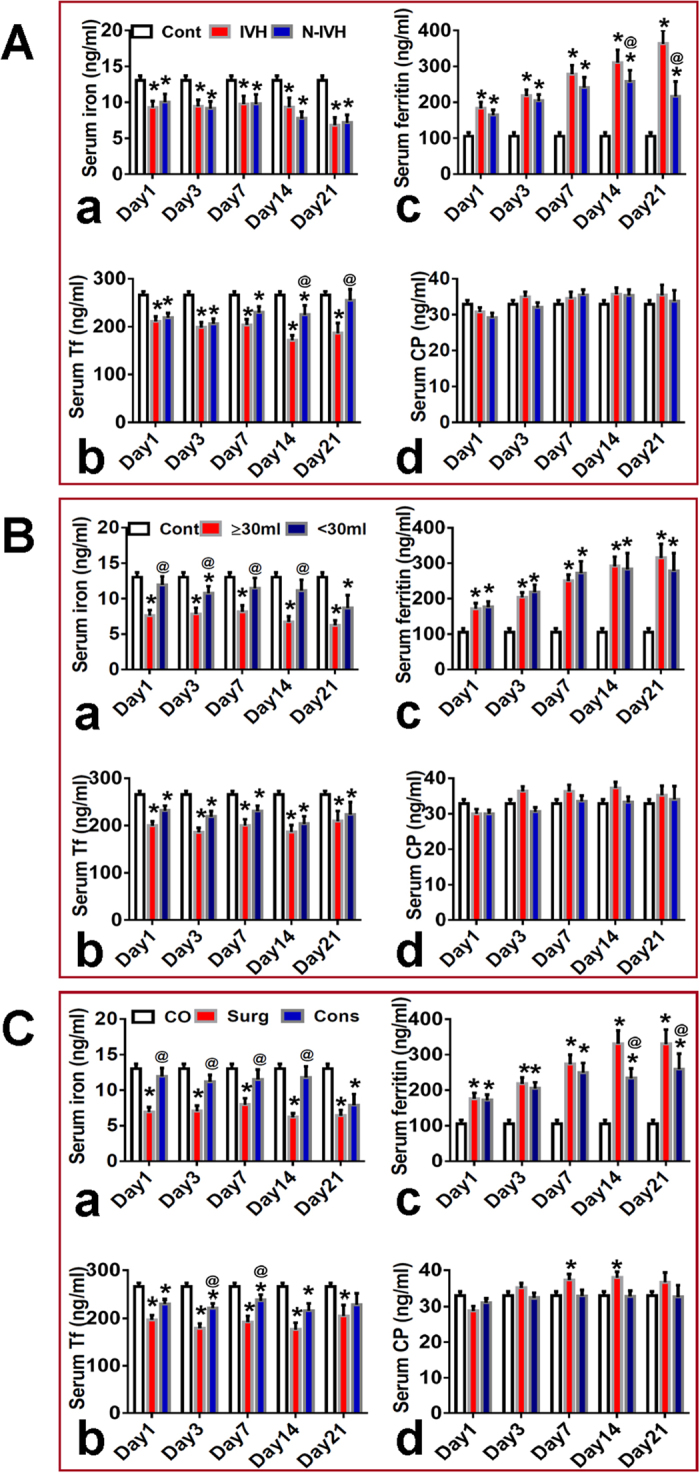
Effects of intraventricular hemorrhage, hematoma volumes and surgery treatment on the contents of iron, ferritin, transferrin and ceruloplasmin in the serum. Serum samples were taken immediately from the control (n = 75) and ICH patients on admission (day 1), at day 3, 7, 14 and 21 after admission. And then the contents of iron (**a**), transferrin (**b**), ferritin (**c**) and ceruloplasmin (**d**) were determined as described in methods section. (**A**) n = 50 (day 1), 46 (day 3), 42 (day 7), 30 (day 14), 18 (day 21) in IVH group, and n = 50 (day 1), 45 (day 3), 36 (day 7), 22 (day 14), 12 (day 21) in N-IVH group. (**B**) n = 53 (day 1), 46 (day 3), 39 (day 7), 29 (day 14), 21(day 21) in “≥ 30ML” group, and n = 47 (day 1), 45 (day 3), 39 (day 7), 23 (day 14), 9 (day 21) in “<30ML” group. (**C**). n = 46 (day 1), 42 (day 3), 38 (day 7), 29 (day 14), 19 (day 21) in surgery (Surg) group, and n = 54 (day 1), 49 (day 3), 40 (day 7), 22 (day 14), 11 (day 21) in conservative (Cons) group. Data were presented as Mean ± SD. *P < 0.05 vs. Control; ^@^P < 0.05 vs. PO, ≥30 ML or Surgery.

We also compared the changes in the contents of serum iron, ferritin, Tf and CP in patients with (IVH) or without intraventricular hemorrhage (No-IVH), larger (≥30 ml) or smaller (<30 ml) hematoma volume, having received surgery (Surg-P) or conservative treatments (Cons-P). The patients with IVH and No-IVH both were found to have a significantly lower content of iron (Fig. 4A-a) and Tf (Fig. 4A-b) as compared to the control. The levels of serum iron in the IVH patients did not differ from those in the No-IVH patients at all time-points. Also, no significant differences were found in serum Tf between the patients with IVH and No-IVH at day 1, 3 and 7, but not at day 14 and 21 when this indictor in IVH patients was significantly lower than that in No-IVH patients. However, serum ferritin levels in the No-IVH patients were lower than those in the IVH patients although the levels in both IVH and No-IVH groups were significantly higher than those in the control at all time-points (Fig. 4A-c). The significance in the difference was found in serum ferritin levels between the patients with IVH and No-IVH at day 14 and 21. We did not find any differences in serum CP among the control, IVH and No-IVH patients. (Fig. 4A-d)

Hematoma, no matter larger (≥30 ml) or smaller (<30 ml) volumes, was found to be associated with lower contents of iron and Tf and higher levels of ferritin in serum (Fig. 4B). Serum iron (Fig. 4B-a) in the patients with larger hematoma volume were significantly lower than those with smaller hematoma volume at almost all time-points, but no significant differences were found in Tf (Fig. 4B-b) and ferritin (Fig. 4B-c) between the patients with larger and smaller hematoma volumes. In addition, serum iron levels in Surg-P or Cons-P were found to be significantly lower than those in the control at all time-points, and also, iron contents in Surg-P were significantly lower than those in Cons-P at day 1, 3, 7 and 14 (Fig. 4C-a). No significant differences in serum Tf were found between Surg-P and Cons-P although serum Tf in these two groups were significantly lower than that in the controls at all time-points (Fig. 4C-b). The Surg-P and Cons-P both have a significantly higher ferritin in serum as compared to the controls. Ferritin levels in Surg-P were found to be significantly higher than those in Cons-P at day 14 and 21, but not at day 1, 3 and 7 (Fig. 4C-c). No significant differences in serum CP were found between the control and patients with larger or smaller hematoma volume (Fig. 4B-d), and Surg-P or Cons-P (Fig. 4C-d).

### Correlation analysis

#### Relative edema volume or ratio and serum iron, ferritin, Tf or CP contents

CT scans were conducted on admission and also at day 3 or 4 after admission, and hematoma and edema (absolute and relative edema) volumes were measured in 28 patients with supratentorial ICH (site of hemorrhage of 6 patients in Lobar and 22 in Deep location) who had no IVH and did receive surgery treatments. The hematoma, absolute and relative edema volumes of 28 patients at admission and 3 to 4 days afterwards were 19.26 ± 11.16 & 23.24 ± 13.74, 21.43 ± 14.58 & 40.71 ± 23.33 and 1.23 ± 0.84 & 2.04 ± 1.18; respectively. Hematoma size increased by approximately 20% from baseline to day 3 to 4, and the absolute edema volume almost doubled during this time period.

**Figure 5:**
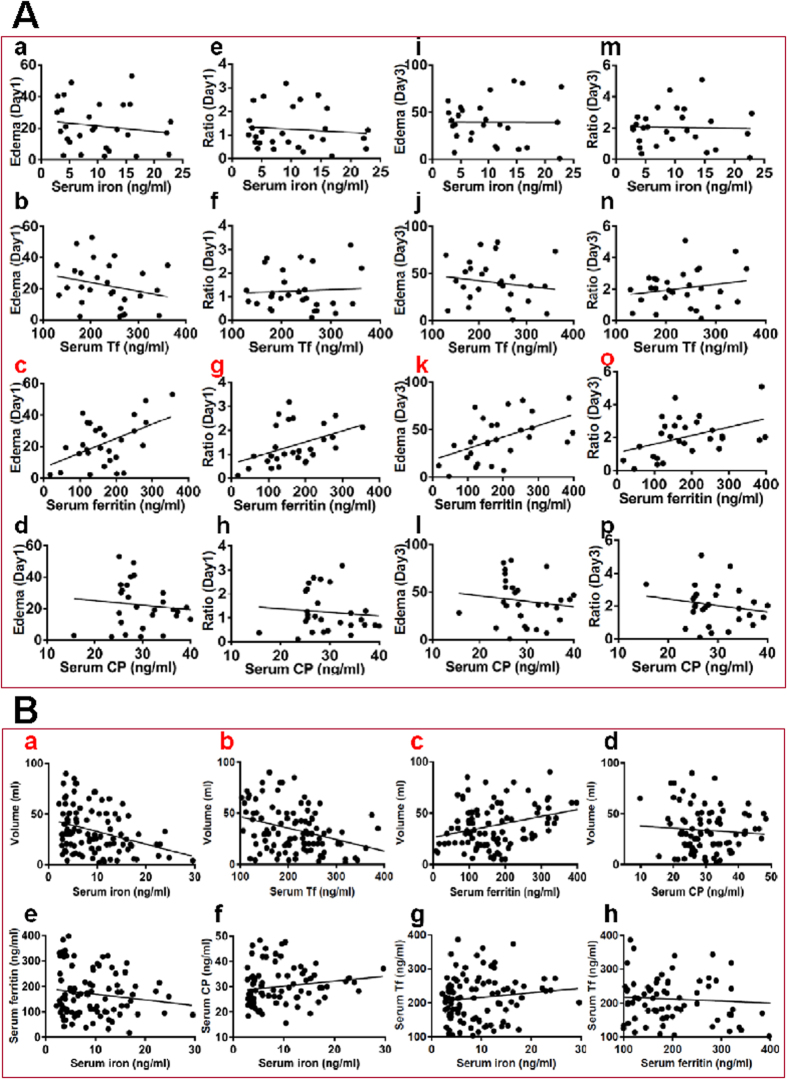
(**A**) Correlation analysis of the relationship between relative edema volume (**a–d**,**i–l**) or ratio (**e–h**,**m–p**) and serum iron, ferritin, transferrin, or ceruloplasmin collected from 28 supratentorial ICH patients who had no IVH and did receive surgery treatments on admission (day 1) (**a–h**) and at day 3 (**i–p**) after admission. (**B**) Correlation analysis of the relationship between hematoma volumes and serum iron (**a**), transferrin (**b**), ferritin (**c**) or ceruloplasmin (**d**) contents, between serum iron and ferritin (**e**), ceruloplasmin (**f**) or transferrin (**g**), and between ferritin and transferrin (**h**) contents collected on admission (day 1) in 100 patients.

The relationship between relative edema volume (Absolute edema - Hematoma) or ratio ([Absolute edema - hematoma] / Hematoma) and serum iron, ferritin, Tf or CP was determined by plotting the values for the relevant pairs against one another as described previously^17^. Figure 5A listed the results of correlation analysis. Highly significant correlations were found between relative edema volume and ferritin (Fig. 5A-c), relative edema ratio and ferritin at day 1 (Fig. 5A-g) and day 3 (Fig. 5A-k-o).

#### Hematoma volume and serum iron, ferritin, Tf or CP contents

We also used Spearman correlation coefficient to determine whether there is a correlation between hematoma volume and serum iron, ferritin, Tf or CP contents collected at day 1 in 100 patients. Figure 5B listed the results of correlation analysis. Highly significant correlations were found between hematoma volume and serum iron (Fig. 5B-a), Tf (Fig. 5B-b) or ferritin (Fig. 5B-c).

#### Serum iron and serum ferritin, CP or Tf, and serum ferritin and serum Tf contents

Finally, the relationship between serum iron and ferritin, CP or Tf, and ferritin and Tf contents collected at day 1 in 100 patients was also determined by using Spearman correlation coefficient. Figure 5B-e-h listed the results of correlation analysis. No significant correlations were found between variables.

## Discussion

In the present study, we first investigated the changes in serum iron and three main iron-handling proteins ferritin, Tf and CP in 100 consecutive patients with acute spontaneous ICH during the period from admission (day 1) to day 21, and then analyzed effects of intraventricular hemorrhage, hematoma volumes and surgery treatment on the contents of iron, ferritin, Tf and CP in the serum, and finally conducted correlation analysis between relative edema volume, ratio or hematoma volume and serum iron, ferritin, Tf or CP contents, and serum iron and one of three main iron-handling proteins.

We demonstrated for the first time that serum ferritin increased significantly with time from admission until day 21 and that serum ferritin in patients with the poor- and good-outcome were both significantly higher than those in the control at all time-points. We also found that there were significant differences in serum ferritin between patients with the poor- and good-outcome at day 1 and 7 when serum ferritin in the former were significantly higher than that in the latter. Analysis of the distribution of mRS scores at 90 days by baseline levels of ferritin categorized in quartile showed that the higher the ferritin quartile, the worse the distribution of mRS scores. In addition, correlation analysis demonstrated that there were highly significant correlations between serum ferritin and relative edema volume as well as ratio in 28 patients with supratentorial ICH at day 1 and day 3 and also between serum ferritin and hematoma volume in 100 patients at day 1. These findings were consistent with the results reported by other groups, providing further evidence that serum ferritin measured at day 3 to 4 after ICH were associated with relative perihematoma edema volume^8^,^13^,^14^ and those on admission could predict clinical outcome in patients with ICH^15^,^16^.

The increased total serum iron levels have been observed in rat models after experimental ICH^18^, and also the measurements of this indicator in serum are easy to be conducted in clinic. However, it is really surprising that not much information about the changes in serum iron in patients with ICH is available, except for a recent study which was conducted in the patients with acute hemorrhagic stroke using atomic absorption spectrophotometry^19^. Tf is unique in chelating iron with very high affinity. It has been reported that Tf concentration is significantly increased in cerebral spinal fluid after subarachnoid hemorrhage^20^. It has also been demonstrated that Tf could protect neurons from an iron-dependent injury in an established cell culture model of hemoglobin neurotoxicity^21^. However, at present, there is no report on the effects of ICH on serum Tf in patients with ICH.

In the present study, therefore we also investigated the changes of serum iron and Tf in patients with ICH and demonstrated for the first time that both serum iron and Tf levels at day 1 (admission) in patients with poor-outcome were significantly lower than those in the patients with good-outcome as well as in the control. The findings implied that the significantly lower iron and Tf on admission might be related to the poorer outcome and also suggested that serum iron and Tf, the same as ferritin, both could be considered as the important predictors of clinical outcome in patients with ICH. Based on the findings in this study plus the previous studies^15^,^16^, we proposed that a combination of serum iron, ferritin and Tf is a reliable predictor of functional outcome in patients with ICH compared with ferritin only. Also, correlation analysis showed that there were highly significant correlations between serum iron or Tf and hematoma volume in 100 patients at day 1. This might suggested that both serum iron and Tf are negatively associated with the size of hematoma volume.

Compared with the controls, serum iron levels were significantly lower in patients with ICH in the present study, rather than higher as found in patients with acute hemorrhagic stroke^19^ and in rat models of ICH^18^. The opposite results might be partly due to the differences in age (44 ± 6 vs. 56 ± 12) and sex (female/male:12/14 vs. 28/72) of patients and species (patients vs. rats), therefore further investigations about the relevant causes are needed. The present study showed that serum iron in patients with ICH were significantly lower than those in the control not only at day 1 but also other time-points (day 3–21). The data suggested that the response of serum iron to ICH is a chronic or long-term rather than only an acute or short-term reaction. The relevant mechanisms involved in the response are unknown. However, the changes in serum iron induced by ICH should be not related to the responses of ferritin, Tf and CP to ICH because correlation analysis showed no significant correlation between serum iron and these three iron metabolism proteins. The increased iron concentration in ICH area and the inflammation-mediated activation in p-STAT3 pathway^22^ both could induce an increase in hepcidin expression. Therefore, it is highly likely that the reduced iron in the patients with ICH might be partly associated with increased hepcidin. In addition, it is worthy to be investigated whether increasing iron concentration in serum, by affecting the relevant iron metabolism proteins (i.e., inhibiting hepcidin expression) rather than direct-iron supplement, is beneficial to improve functional outcome.

Ceruloplasmin is a key protein involved in cell iron transport^23^,^24^,^25^ and also an acute phase reactant with a number of functions including ferroxidase activity^26^,^27^, anti-oxidant functions and the prevention of the formation of free radicals^28^, and inhibition of neutrophil myeloperoxidase^29^. This protein has also been suggested to have a protective role for brain from iron-induced injury following subarachnoid hemorrhage^30^. Therefore, we investigated the changes in this protein in ICH patients. In contrast to what we expected, CP levels showed no significant changes in the patients with ICH as compared to the controls and did not correlate with any of the outcomes studied in the present study. This suggested that CP seems unlikely to play a major role in the body’s response to ICH.

We also examined the effects of intraventricular hemorrhage, hematoma volumes and surgery treatment on the contents of iron, ferritin, Tf and CP in the serum. We did not find any differences in serum iron between ICH patients with or without IVH at any-time points, suggesting that IVH has no significant effect on serum iron. Also, no significant differences in serum ferritin and Tf were found between ICH patients with or without IVH at day 1–7, but not at day 14 and 21, implying that IVH could affect serum ferritin and Tf levels at the late stage of ICH. Serum iron, but not Tf, in ICH patients with larger (≥30 ml) hematoma volume or having received surgery treatments was significantly lower than that in ICH patients with smaller (<30 ml) hematoma volume or having received conservative treatments at day 1–14; respectively, implying that hematoma volume and surgery treatment both can affect serum iron, but not serum Tf.

No significant differences in serum ferritin were found between the ICH patients with larger or smaller hematoma volume at all time-points, and having received surgery or conservative treatments at day 1–7, but not at day 14 and 21, implying that surgery treatment might have an effect on serum ferritin at late stage. No differences were found in serum CP between the ICH patients with or without IVH, with larger or smaller hematoma volume, and having received surgery or conservative treatments; respectively, showing that serum CP is not sensitive to these factors.

## Conclusions

To our knowledge, the present study is the first investigation to examine and analyze the effects of ICH on serum iron and iron-handling proteins ferritin, Tf and CP and relationship of these biomarkers of iron metabolism in serum with outcome in patients with ICH at day 1–21. Firstly, we showed that higher stroke severity, lower platelet concentration in the blood, deep location of hematoma, subtentorial hematoma and intraventricular extension of hemorrhage at day 1 (baseline) were associated with poor clinical outcome. Secondly, we provided further evidence that serum ferritin contents were associated with relative hematoma edema volume and could predict clinical outcome in patients with ICH. Thirdly, our findings implied that not only the increased serum ferritin but also the reduced serum iron and Tf are associated with functional outcome as well as hematoma volume in patients with ICH. Fourthly, we found that intraventricular hemorrhage, hematoma volumes and surgery treatments have different associations with the contents of iron, ferritin, Tf and CP in the serum at different stages after ICH in patients. Finally, serum CP levels were found to have no significant changes in the patients with ICH, suggesting that CP seems unlikely to play a major role in the body’s response to ICH. Further studies on the effects of ICH on hepcidin expression and the relevant mechanisms are needed.

## Methods

### Subjects

A total of 100 consecutive patients with acute intracerebral hemorrhage (ICH) were enrolled in this study. All patients, aged 40–80 years old, were admitted to the Department of Neurosurgery, The First Affiliated Hospital of Third Military Medical University (Chongqing, China) within 24 h after stroke onset during the period from May 2014 and June 2015. Patients were examined neurologically and the diagnosis was also confirmed by CT scans of the brain. All patients were admitted at an acute stroke unit and treated according to the guidelines of the European Stroke Initiative Writing Committee^31^.

Patients with secondary causes of ICH, such as anticoagulant use, underlying aneurysm vascular malformation or tumor, head trauma, or hemorrhagic transformation of ischemic infarcts were excluded. Patients were also excluded if they had an underlying medical disease that had an effect on the level of iron and ferritin such as anemia and severe liver or renal disease, intravenous drug abuse and pregnancy.

A control group comprised of 75 age- and sex-matched healthy individuals were recruited from volunteers. They were asymptomatic with unremarkable medical histories and normal physical examination results and had blood drawn solely for study participation. Informed consent was signed by all subjects before the collection of information. The study was approved by the Ethical Committee of The first Affiliated Hospital of Third Military Medical University, PLA. ClinicalTrials.gov Identifier: NCT02135783 (May 7, 2014). The methods were carried out in accordance with the approved guidelines.

### Data Collection

On admission (day 1), we collected demographic, clinical, laboratory, and radiological data, including: age, sex, hemorrhage onset-to-imaging time, the history of alcohol use and habitual smoking, body temperature, arterial blood (systolic and diastolic) pressure, serum glucose, complete blood counts (hemoglobin, hematocrit, white cell count, red cell count and platelet) and ICH scores^32^. Stroke severity was quantified by using the Glasgow Coma Scale (GCS)^33^ and the National Institute of Health Stroke Scale (NIHSS)^34^ on admission, at day 3, 7, 14 and 21 after admission. Neurological improvement was defined as any improvement in GCS and NIHSS score points at follow-up compared to the scores on admission.

### Radiological measurements

In addition to being conducted for the diagnosis of ICH on admission, CT scans were done between 3 and 4 days after admission. All CT scans were centrally evaluated by an experienced investigator who was masked to patients’ clinical and biochemical data. Volumetric measurements of ICH and edema (absolute and relative) were conducted and locations for ICH determined according to Kothari *et al.*^18^ and Mehdiratta *et al.*^8^. ICH hematoma volume was estimated by using ABC/2 method^18^.

### Laboratory determinations

Serum samples were taken immediately on admission (within 5 h of symptom onset), at day 3, 7, 14 and 21 after admission and then stored at *−*80^o^C for the determination of the changes in serum iron, ferritin, transferrin and ceruloplasmin contents. Serum ferritin levels were determined by electrochemiluminescence immunoassay using an analyzer ELECSYS 2010 (Roche Diagnostics GmbH), and serum iron, Tf and CP contents were measured as described previously^18^,^35^,^36^,^37^. These measurements were completed in our central laboratory by investigators blinded to the clinical outcome and all relevant findings. Clinical investigators were unaware of the laboratory results until the end of the study.

### Evaluation of outcome

Functional outcome was evaluated using the modified Rankin scale (mRS)^38^ at 90 days following treatment initiation (a follow-up period of 90 days) performed by a designated neurologist who was blinded to treatment tier. The poor outcome was defined as mRS score ≥ 3 and the good-outcome as mRS score < 3.

### Statistical analysis

All data were presented as mean ± standard deviation (SD). Statistical analyses were performed using SPSS software for Windows (version 13.0) (SPSS, Inc., Chicago, IL). The difference among different groups was determined by one-way or two-way analysis of variance (ANOVA) in appropriate studies followed by a post hoc Newman-Keuls for multiple comparisons. Spearman correlation coefficient was used to determine the presence or absence of a correlation between variables. A probability value of less than 0.05 was taken to indicate statistically significant.

## Additional Information

**How to cite this article**: Yang, G. *et al.* A combination of serum iron, ferritin and transferrin predicts outcome in patients with intracerebral hemorrhage. *Sci. Rep.* **6**, 21970; doi: 10.1038/srep21970 (2016).

## Figures and Tables

**Table 1:** Clinical, Biochemical and Radiological Characteristics by Outcome Groups.

	Poor OC (n = 59)	Good OC (n = 41)	P Value
Male (%)	42 (71.2)	30 (73.1)	0.828
Age (year)	56 ± 12	55 ± 8	0.806
Time from onset to admission (h)	8 [2–24]	10 [3–24]	0.211
History of vascular risk factors (%)			
History of hypertension	42 (60.8)	25 (60.9)	0.286
History of diabetes mellitus	9 (15.2)	3 (7.3)	0.374
History of coronary heart disease	4 (6.7)	1 (2.4)	0.608
History of stroke	10 (16.9)	5 (12.1)	0.513
Alcohol consumption	17 (28.8)	11 (26.8)	0.787
Smoke habit (current)	25 (42.3)	15 (36.6)	0.516
Vital signs			
Body temperature (°C)	36.6 ± 0.4	36.8 ± 0.6	0.055
Systolic blood pressure (mmHg)	173 ± 25	163 ± 27	0.056
Diastolic blood pressure (mmHg)	96 ± 14	96 ± 24	0.906
GCS at baseline 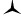	9 [5–15]	14 [5–15]	<0.001
NIHSS at baseline 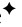	16 [5–22]	6 [0–18]	<0.001
ICH at baseline 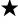	2 [0–4]	1 [0–3]	<0.001
CT imaging data			
Left hemisphere hematoma	32 (54.2)	24 (58.5)	0.670
Lobar hematoma	5 (8.5)	9 (21.9)	0.056
Deep location hematoma	54 (91.5)	27 (65.9)	<0.001
Subtentorial hematoma	0 (0.0)	5 (12.2)	<0.010
Intraventricular extension of hemorrhage	35 (59.3)	15 (36.5)	<0.025
Laboratory data			
Erythrocyte (10E^12^/L)	4.6 ± 0.6	4.6 ± 0.6	0.592
Hemoglobin (g/L)	139.5 ± 22.2	138.2 ± 18.0	0.754
Leukocyte, 10E^9^/L	13.7 ± 7.5	9.7 ± 3.1	0.153
Platelet (10E^9^/L)	172.6 ± 59.0	200.3 ± 66.1	<0.050
Serum glucose (mmol/L)	8.8 ± 2.2	7.3 ± 2.7	0.062
Fibrinogen (g/L)	2.9 ± 1.7	2.7 ± 1.5	0.637
Days in ICU	10 ± 9	6 ± 4	<0.010
Days in Hospital	19 ± 12	20 ± 9	0.360
Surgery	38 (64.4)	8 (19.5)	<0.001
Tracheotomy	18 (30.5)	1 (2.4)	<0.001
Death in Hospital	4 (6.8)	0 (0.0)	0.142
Death in 90-Day	14 (23.7)	0 (0.0)	<0.001

Data are expressed as n (%), mean ± SD, or median [quartiles] as appropriate. 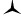
Scores on the GCS (Glasgow Coma Scale) range from 15 (fully conscious) to 3 (deep coma); 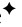
Scores on the NIHSS (National Institutes of Health Stroke Scale) range from 0 (normal neurologic status) to 42 (coma with quadriplegia); 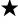
ICH, Components of the ICH score include age, GCS score, ICH hematoma volume, ICH hematoma location (supratentorial or infratentorial), and presence of IVH, Scores on the ICH range from 0 to 6; Deep location refers to location in the basal ganglia or thalamus; Lobar refers to location in the temporal, frontal, or occipital. ICU, Intensive Care Unit.
